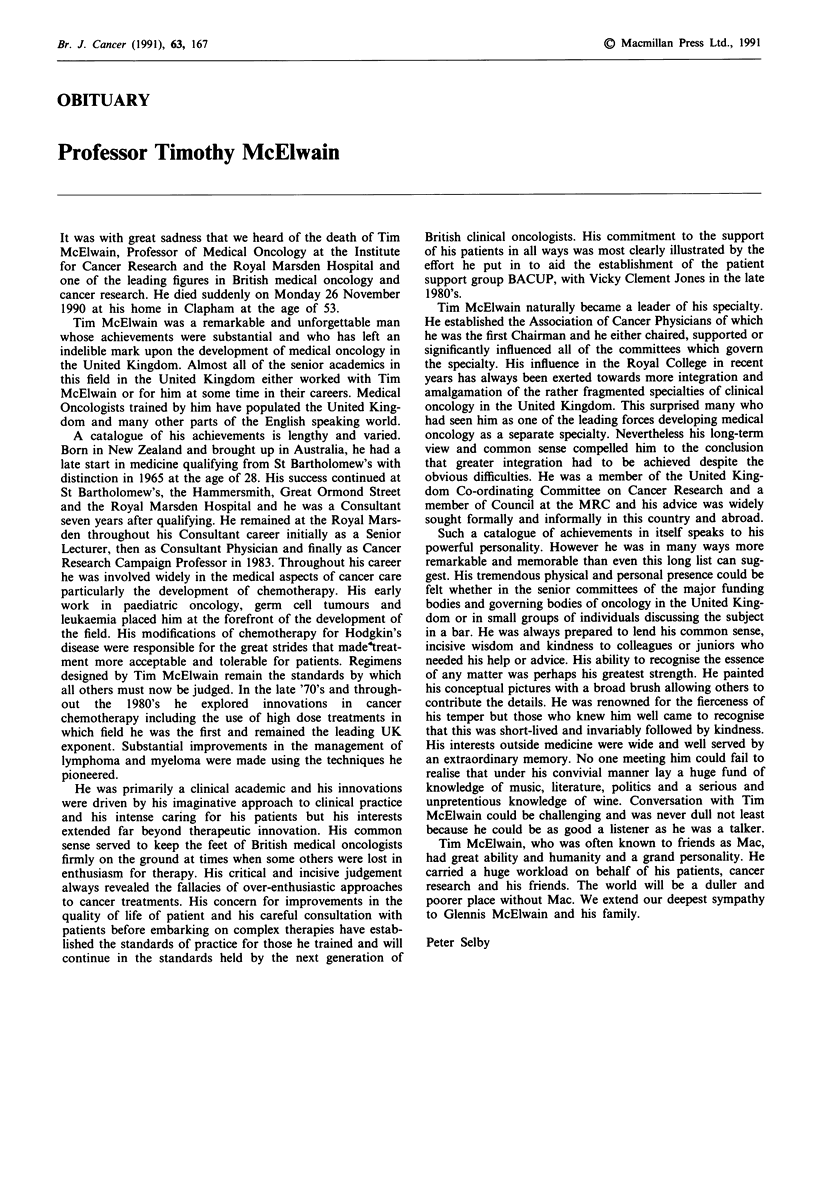# Professor Timothy McElwain

**Published:** 1991-02

**Authors:** Peter Selby


 © Macmillan Press Ltd., 1991

### OBITUARY

### Professor Timothy McElwain

It was with great sadness that we heard of the death of Tim McElwain, Professor of Medical Oncology at the Institute for Cancer Research and the Royal Marsden Hospital and one of the leading figures in British medical oncology and cancer research. He died suddenly on Monday 26 November 1990 at his home in Clapham at the age of 53.

Tim McElwain was a remarkable and unforgettable man whose achievements were substantial and who has left an indelible mark upon the development of medical oncology in the United Kingdom. Almost all of the senior academics in this field in the United Kingdom either worked with Tim McElwain or for him at some time in their careers. Medical Oncologists trained by him have populated the United Kingdom and many other parts of the English speaking world.

A catalogue of his achievements is lengthy and varied. Born in New Zealand and brought up in Australia, he had a late start in medicine qualifying from St Bartholomew's with distinction in 1965 at the age of 28. His success continued at St Bartholomew's, the Hammersmith, Great Ormond Street and the Royal Marsden Hospital and he was a Consultant seven years after qualifying. He remained at the Royal Marsden throughout his Consultant career initially as a Senior Lecturer, then as Consultant Physician and finally as Cancer Research Campaign Professor in 1983. Throughout his career he was involved widely in the medical aspects of cancer care particularly the development of chemotherapy. His early work in paediatric oncology, germ cell tumours and leukaemia placed him at the forefront of the development of the field. His modifications of chemotherapy for Hodgkin's disease were responsible for the great strides that made treatment more acceptable and tolerable for patients. Regimens designed by Tim McElwain remain the standards by which all others must now be judged. In the late '70's and throughout the 1980's he explored innovations in cancer chemotherapy including the use of high dose treatments in which field he was the first and remained the leading UK exponent. Substantial improvements in the management of lymphoma and myeloma were made using the techniques he pioneered.

He was primarily a clinical academic and his innovations were driven by his imaginative approach to clinical practice and his intense caring for his patients but his interests extended far beyond therapeutic innovation. His common sense served to keep the feet of British medical oncologists firmly on the ground at times when some others were lost in enthusiasm for therapy. His critical and incisive judgement always revealed the fallacies of over-enthusiastic approaches to cancer treatments. His concern for improvements in the quality of life of patient and his careful consultation with patients before embarking on complex therapies have established the standards of practice for those he trained and will continue in the standards held by the next generation of British clinical oncologists. His commitment to the support of his patients in all ways was most clearly illustrated by the effort he put in to aid the establishment of the patient support group BACUP, with Vicky Clement Jones in the late 1980's.

Tim McElwain naturally became a leader of his specialty. He established the Association of Cancer Physicians of which he was the first Chairman and he either chaired, supported or significantly influenced all of the committees which govern the specialty. His influence in the Royal College in recent years has always been exerted towards more integration and amalgamation of the rather fragmented specialties of clinical oncology in the United Kingdom. This surprised many who had seen him as one of the leading forces developing medical oncology as a separate specialty. Nevertheless his long-term view and common sense compelled him to the conclusion that greater integration had to be achieved despite the obvious difficulties. He was a member of the United Kingdom Co-ordinating Committee on Cancer Research and a member of Council at the MRC and his advice was widely sought formally and informally in this country and abroad.

Such a catalogue of achievements in itself speaks to his powerful personality. However he was in many ways more remarkable and memorable than even this long list can suggest. His tremendous physical and personal presence could be felt whether in the senior committees of the major funding bodies and governing bodies of oncology in the United Kingdom or in small groups of individuals discussing the subject in a bar. He was always prepared to lend his common sense, incisive wisdom and kindness to colleagues or juniors who needed his help or advice. His ability to recognise the essence of any matter was perhaps his greatest strength. He painted his conceptual pictures with a broad brush allowing others to contribute the details. He was renowned for the fierceness of his temper but those who knew him well came to recognise that this was short-lived and invariably followed by kindness. His interests outside medicine were wide and well served by an extraordinary memory. No one meeting him could fail to realise that under his convivial manner lay a huge fund of knowledge of music, literature, politics and a serious and unpretentious knowledge of wine. Conversation with Tim McElwain could be challenging and was never dull not least because he could be as good a listener as he was a talker.

Tim McElwain, who was often known to friends as Mac, had great ability and humanity and a grand personality. He carried a huge workload on behalf of his patients, cancer research and his friends. The world will be a duller and poorer place without Mac. We extend our deepest sympathy to Glennis McElwain and his family.

Peter Selby